# Clinical use of autologous adipose-derived stromal vascular fraction cell injections for hip osteoarthritis

**DOI:** 10.1016/j.reth.2023.06.006

**Published:** 2023-06-15

**Authors:** Yuma Onoi, Tomoyuki Matsumoto, Satoshi Sobajima, Masanori Tsubosaka, Shinya Hayashi, Takehiko Matsushita, Hideki Iwaguro, Ryosuke Kuroda

**Affiliations:** a Department of Orthopaedic Surgery, Kobe University Graduate School of Medicine, Kobe, Japan; b Department of Orthopaedic Surgery, Sobajima Clinic, Osaka, Japan

**Keywords:** Adipose-derived stromal vascular fraction cells, Stem cell therapy, Hip osteoarthritis, Conservative treatment

## Abstract

**Introduction:**

Currently, studies on adipose-derived stromal vascular fraction (SVF) cells are attracting increasing attention because they have the potential to differentiate into a subset of cell types, such as bone marrow-derived mesenchymal stromal cells (MSCs), and are easier to harvest than MSCs, thus making them easier to apply clinically. This study evaluated the short-term clinical outcomes of SVF cell therapy for hip osteoarthritis (OA).

**Methods:**

Forty-two patients were enrolled in this study; these patients received a single injection comprising an average of 3.8 (standard deviation [SD], ±1.3) × 10^7^ SVF cells into the hip joint. All patients were followed-up for at least 6 months. The mean age of the patients was 60.2 years (SD, ±9.4 years). Kellgren–Lawrence (KL) grades II, III, and IV based on radiography were 13, 13, and 16 patients, respectively. SVF cells were obtained from the subcutaneous fat of the abdomen or breech using a Celution® 800/CRS system. The average cell viability of SVF cells was 90.8% (SD, ±2.8%). Clinical assessments were performed using the Harris Hip Score (HHS), Japanese Orthopaedic Association Hip Disease Evaluation Questionnaire (JHEQ) score, and visual analog scale (VAS) score to evaluate pain. Images were evaluated using radiography, and T2 mapping values were obtained using a 1.5-T magnetic resonance imaging system. These clinical and imaging assessments were followed from preoperatively to 6 months postoperatively.

**Results:**

The HHS, JHEQ score, and VAS score improved significantly from 22.5 (SD, ±16.6), 26.6 (SD, ±11.3), and 75.5 (SD, ±15.8) preoperatively to 46.8 (SD, ±27.2), 39.4 (SD, ±19.7), and 46.5 (SD, ±27.9), respectively, at 6 months postoperatively. KL grade II showed significant improvement in clinical outcome from preoperative to postoperative, while KL grade IV showed slight or little improvement. The center edge angle, acetabular head index on the radiographs, and T2 mapping values did not change significantly from preoperatively to 6 months postoperatively.

**Conclusions:**

SVF cell injection in the hip joint showed good short-term clinical efficacy for reducing hip OA symptoms. SVF cell therapy is thus an innovative and effective treatment for hip OA.

## 1 Introduction

Osteoarthritis (OA) results in chronic pain and functional limitations and causes long-term impairment of the elderly. Furthermore, OA is one of the most frequent joint disorders. The hip joint is a weight-bearing joint; after the knee joint, the hip joint is the second most susceptible to OA. Currently, the risk of symptomatic hip OA in the elderly is 25% [1,2]. Aging, overweight status, and lack of exercise cause hip OA and its complications, which are associated with significant social and economic consequences [3]. Traditional conservative treatments for hip OA include nonpharmacological therapies (e.g., activity reduction, weight loss, physical support, and physical therapy) and oral, local, and intra-articular pharmacological therapies (e.g., analgesics, steroids, and nonsteroidal anti-inflammatory drugs) [[4], [5], [6]]. One of the most critical limitations of these therapies is their unpredictable effects, side effects, and inability to treat OA progression [7].

Stem cell therapy has gained attention as a novel treatment for OA. Adipose-derived stromal vascular fraction (SVF) cells comprise a wide variety of cell types, including mesenchymal stromal cells (MSCs), blood cells, macrophages, fibroblasts, pericytes, endothelial cells, smooth muscle cells and their progenitors [8,9]. It is easier to collect SVF cells than other stem cells, such as bone marrow-derived MSCs or adipose tissue-derived MSCs [10]. SVF cells can be harvested in large numbers from autologous adipose tissue and utilized without culturing or differentiation enabling one-step regenerative therapy [11]. Studies have suggested that the safety and efficacy of SVF cells are equal to those of other stem cells in several settings, such as hypertrophic scar remodeling, nerve regeneration, acute myocardial infarction treatment, and cartilage regeneration in some animal models [[12], [13], [14], [15]]. In orthopaedic clinical settings, beneficial effects of SVF cell therapy for OA, especially knee OA, have been observed [16,17]. However, no published clinical studies with adequate sample sizes have evaluated the clinical outcomes of SVF cell therapy for hip OA. Therefore, we designed a prospective case series of intra-articular autologous SVF cell injections for hip OA. We hypothesized that SVF therapy for hip OA would improve clinical outcomes and regenerate articular cartilage.

## 2 Methods

### 2.1 Study design and subject enrollment

This prospective case study aimed to assess the efficacy, feasibility, and safety of autologous SVF cell therapy for patients with hip OA. Degenerative grades of hip OA were assessed from radiographic findings using the Kellgren–Lawrence (KL) classification. Patients with all KL grades were included in this study. The study protocol complied with the Declaration of Helsinki and was approved by the appropriate ethics committees. All patients provided informed consent before participation.

The inclusion criteria were as follows: diagnosed with hip OA at any age; considerable pain and functional decline; failed conservative treatment such as physical therapy, pharmacotherapy, and intra-articular injection of hyaluronic acid or steroids; and provided written informed consent. The exclusion criteria were as follows: preoperative radiographs showing a severe bone loss or dislocation; history of hip injury requiring surgery; active or previous infection of the hip joint; and history of severe disease (such as systemic inflammatory conditions and vascular alterations).

### 2.2 Treatment process

**Fig. 1:**
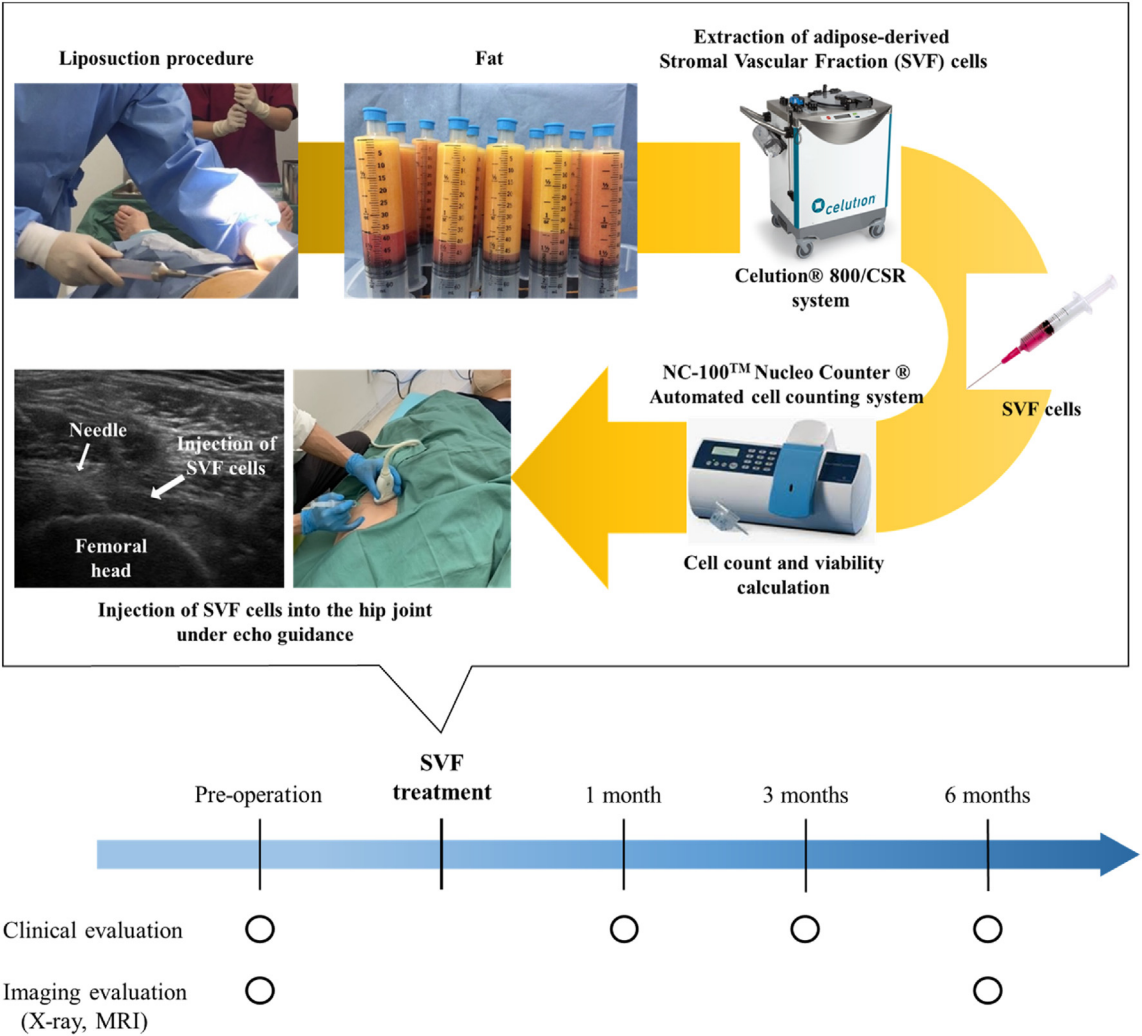
Schema of treatment procedures.

The Celution® 800/CRS system (Cytori Therapeutics Inc., San Diego, CA, USA) was used to obtain SVF cells from the subcutaneous fat of the abdomen or hindquarters. The tissue was washed and degraded utilizing this system to obtain cell concentration. All patients underwent liposuction under general anesthesia. After that, 120–350 mL of adipose tissue was harvested and processed using the Celution® 800/CRS System according to the manufacturer's instructions. Briefly, the tissue was cleaned to remove the blood and remnants. Next, the aspirated adipose tissue was digested by adding Celase® GMP (Cytori Therapeutics Inc.) containing highly purified collagenase and neutral protease enzymes and incubated at 37 °C for 20 min. Thereafter, the SVF cells were concentrated by centrifugation, washed to remove the Celase® reagent, extracted from the system, counted, and prepared in a prescribed volume of 5 mL in lactated Ringer's solution. The entire system can be handled aseptically using saline or lactated Ringer's solution and a single-use Celution™ disposable kit. The SVF cell count and viability were measured using the NC-100™ NucleoCounter® Automated Cell Counting System (Chemometec, Allerod, Denmark), an image cytometer using fluorescent iodide dye. The viable cell membrane was dissolved by mixing the reagent with the sample, and the cell nuclei were easily stained with propidium iodide so that the total cell counts could be determined. However, nonviable cells were directly stained with propidium iodide and counted because they were permeable without processing. Therefore, the viability of SVF cells can be calculated using the total number of cells and the number of nonviable cells (Fig. 1).

At least 2.5 × 10^7^ SVF cells were injected intra-articularly into each joint according to the previously described guidelines [18]. The intra-articular injection of SVF cells in the hip joint was performed with a catheterized needle under echo guidance without anesthesia [19]. After SVF administration, patients were asked to attend the hospital regularly for rehabilitation by a physical therapist and to perform daily home exercises according to the hospital's standardized rehabilitation protocols. Rehabilitation consists of hip range of motion practice, muscle strengthening exercises mainly for hip abductors and quadriceps, and athletic endurance activities such as walking.

### 2.3 Endpoints

The primary endpoint of the present study was an improvement in the clinical assessment scores of the treated patients. The range of motion (ROM) of hip flexion and abduction was measured three times using a goniometer, and the averages were recorded. The hip flexion and abduction muscle force were measured using a handheld dynamometer. Hip flexor strength was measured in the seated position with the hip and knee flexed at 90°. Hip abductor strength was tested in the supine position with the hip and knee fully extended. A handheld dynamometer was placed above the knee. Hip flexor strength was measured by holding the hip in flexion for 3 s. Hip abductor strength was measured by holding the hip in abduction for 3 s. These measurements were performed three times, and the mean values were recorded. Clinical scores included the Harris Hip Score (HHS), Japanese Orthopaedic Association Hip Disease Evaluation Questionnaire (JHEQ) score, and visual analog scale (VAS) score for pain (0–100). The HHS is a clinician-based outcome measure that is traditionally used worldwide to evaluate patients with hip OA and consists of the following subscales: pain (0−44), activities of daily living (0−14), function (0−33), absence of deformity (0–4), and range of movement (0–5). Higher scores indicate better performance [20]. The JHEQ is a patient-oriented evaluation tool developed by the Japanese Orthopaedic Society to assess the hip condition of Asian patients properly. The JHEQ includes items that appropriately assess Asian lifestyle habits, such as deep bending of the knee and hip, sitting on the floor, standing on the floor, and sitting with the legs folded under the thighs. The JHEQ includes the following subscales: pain (0−28), movement (0−28), and mental health (0−28). The total possible score is 84, and higher scores indicate better performance [21].

**Fig. 2:**
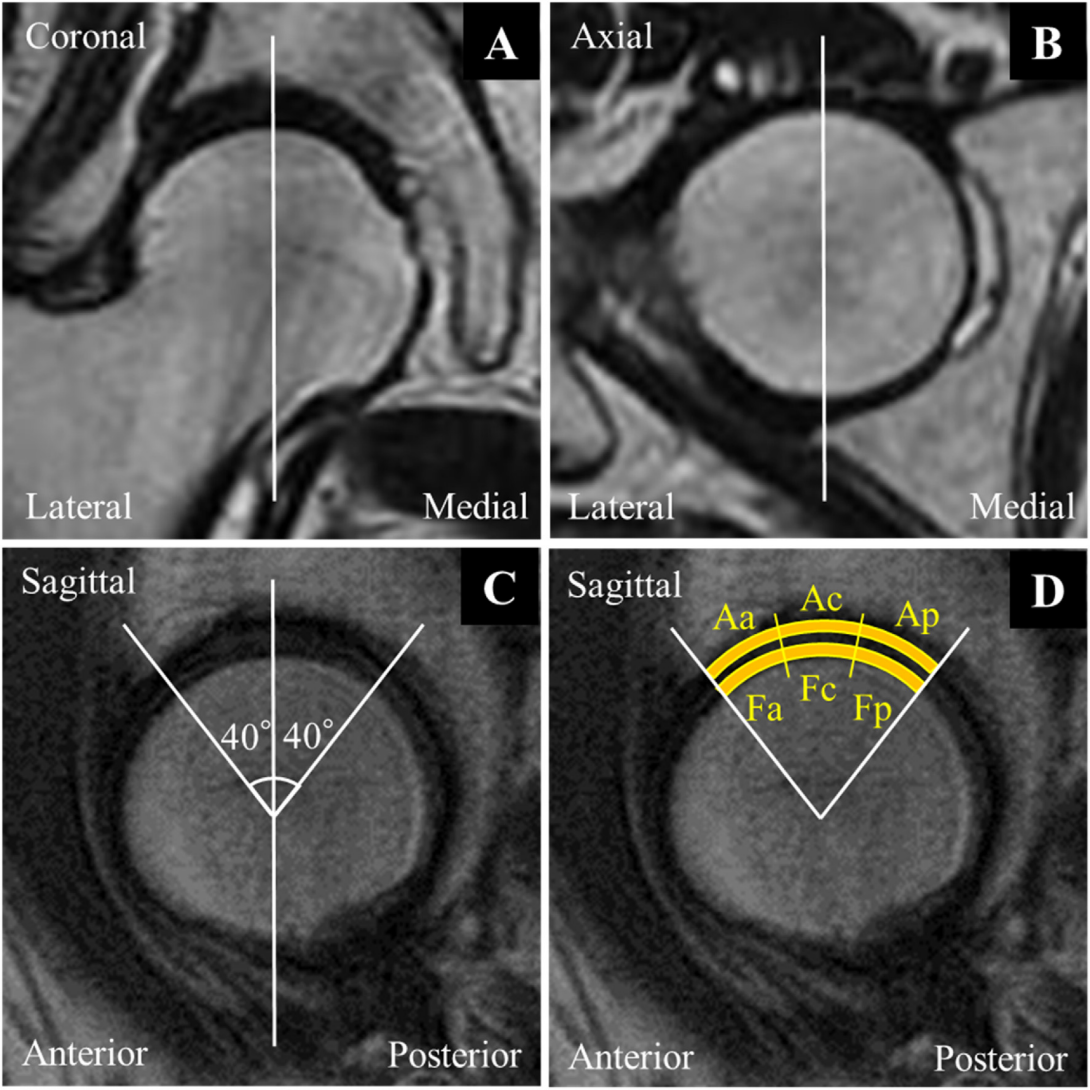
The coronal (A) and axial planes (B). A sagittal plane passing through the center of the femoral head (C) was selected. The region of interest (ROI) was set within 40° anteriorly and 40° posteriorly of a perpendicular line through the center of the femoral head in the sagittal plane (C). The ROI was divided into radial sections with equal widths and defined as the anterior femoral head (Fa), center femoral head (Fc), posterior femoral head (Fp), anterior acetabulum (Aa), center acetabulum (Ac), and posterior acetabulum (Ap) (D).

The secondary endpoint included the imaging appearance, including those of the center edge angle and acetabular head index, determined using radiography [22] and T2 mapping with a 1.5-T magnetic resonance imaging system (Sigma Exite HDx; GE Healthcare, Waukesha, WI, USA) [23]. According to the literature [24], T2 mapping values were calculated as follows: the center of the femoral head was identified using three T2-weighted images of the coronal, axial, and sagittal views (Fig. 2A, B, C). Using the sagittal image, the center of the femoral head was determined using concentric circles, and a vertical line passing through the center point was drawn. The region of interest used to evaluate the hip cartilage was defined as the articular cartilage of the femoral head and acetabulum within 40° anteriorly and 40° posteriorly from the centerline (Fig. 2C and D) to minimize the influence of the magic angle. To assess the variability of individual parts of the articular cartilage T2 values after SVF therapy, the articular cartilage of the femoral head and that of the acetabulum were segmented into the following six sections with equal widths: anterior femoral head, central femoral head, posterior femoral head, anterior acetabular, central acetabular, and posterior acetabular posterior part (Fig. 2D). The average T2 mapping values of all six regions of interest were measured. During this analysis, a lower T2 mapping value indicated a better result and a lower degree of articular cartilage degeneration.

All clinical evaluations were performed by an independent, experienced physical therapist preoperatively and at 1, 3, and 6 months after SVF cell therapy. Imaging evaluations were performed preoperatively and 6 months after SVF cell injection by an independent orthopaedic surgeon with 15 years of experience performing magnetic resonance imaging analyses of the hip joints (Fig. 1). All image measurements were conducted twice. The correlation coefficients for intra-observer reliability were more than 0.80 (range, 0.82–0.96) for all measurements. All adverse events incidence, severity, and consequences were documented as part of the safety evaluation.

### 2.4 Statistical analysis

The results were analyzed using EZR statistical software (Saitama Medical Center, Jichi Medical University, Saitama, Japan) [25], and the values are presented as the mean ± standard deviation (SD). Friedman's test and Bonferroni's correction were used to analyze differences in the ROM, muscle forces, HHS, JHEQ score, and VAS score at the four-time points (preoperatively and 1, 3, and 6 months postoperatively). The Kruskal–Wallis and Steel–Dwass post hoc tests were performed to compare clinical outcomes based on the KL classifications. The preoperative imaging assessment values were compared to those at 6 months postoperatively, and the differences were analyzed using the paired *t*-test. Statistical significance was set at P<0.05. A preliminary statistical power analysis was conducted using G power 3 [26]; assuming a medium effect size (effect size=0.40) based on the prespecified significance level (α<0.05), a power (1-β) of 0.8 was necessary. The estimated sample size was 41.

## 3 Results

### 3.1 Patient background

**Fig. 3:**
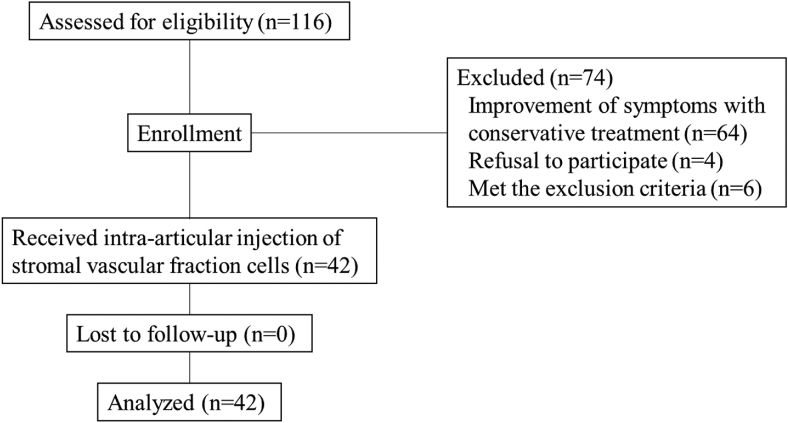
Patient flow diagram.

A total of 116 patients presented to our institution for SVF cell therapy between April 2017 and July 2019. Of these, 64 were excluded because their symptoms had improved with conservative treatments, such as rehabilitation, medication, and intra-articular injection of hyaluronic acid or corticosteroids. Four patients declined to participate in this study. Based on our exclusion criteria, six patients were excluded (three patients with severe bone loss observed using radiography, one with prior hip trauma requiring surgery, one with an active or previous hip joint infection, and one with a history of critical systemic disease). Consequently, 42 patients were enrolled in this study and received an intra-articular SVF cell injection in the hip joint. No participant was lost to follow-up; therefore, all 42 patients (42 hips) participated in the study, and the follow-up rate was 100% (Fig. 3). All eligible patients were followed-up for at least 6 months, with a mean observation period of 2.9 years (SD, ±0.8 years). The mean age at the start of SVF therapy was 60.2 years (SD, ± 9.4 years), and the mean body mass index was 22.3 kg/m^2^ (SD, ±3.4 kg/m^2^). According to the KL classification, patients were separated into the grade I (0 patients), grade II (13 patients), grade III (13 patients), and grade IV (16 patients) groups. The mean preoperative center edge angle was 17.2° (SD, ±12.8°). The mean acetabular head index was 69.0% (SD, ±11.6%) (Table 1). The mean fat aspiration volume, purified SVF cell count, and SVF cell viability were 313.2 mL (SD, ±46.7 mL), 3.8 (SD, ±1.3) × 10^7^, and 90.8% (SD, ±2.8%), respectively.

**Table 1:** Patients’ background.

Characteristics	Baseline Data
Sex (female/male); n (%)	37/5 (88%/12%)
Age (mean ± standard deviation); yrs	60.2 ± 9.4
Body mass index; kg/m^2^	22.3 ± 3.4
Center-edge angle at baseline; degree	17.2 ± 12.8
Acetabular head index; %	69.0 ± 11.6
Kellgren–Lawrence classification; n (%)	
Grade Ⅰ	0 (0%)
Grade Ⅱ	13 (31%)
Grade Ⅲ	13 (31%)
Grade Ⅳ	16 (38%)

### 3.2 Clinical evaluation

The hip abduction ROM was significantly greater at 1, 3, and 6 months postoperatively compared to that preoperatively. Hip flexion muscle strength was significantly greater at 1, 3, and 6 months postoperatively compared to that preoperatively (Table 2).

**Table 2:** Clinical evaluation results.

ROM of the hip					
Flexion	Mean ± S.D.	*P*	Abduction	Mean ± S.D.	*P*
Preoperative	93.1 ± 17.3 (°)		Preoperative	23.3 ± 7.5 (°)	
1 month	94.8 ± 15.8 (°)	0.113	1 month	25.7 ± 7.2 (°)	0.016
3 months	95.1 ± 15.8 (°)	0.169	3 months	25.1 ± 7.4 (°)	0.047
6 months	95.1 ± 15.2 (°)	0.200	6 months	26.2 ± 7.1 (°)	0.006
Muscle force					
Flexion			Abduction		
Preoperative	192.1 ± 70.4 (Nm)		Preoperative	178.7 ± 65.3 (Nm)	
1 month	213.4 ± 74.6 (Nm)	0.009	1 month	158.8 ± 78.2 (Nm)	0.109
3 months	218.3 ± 74.4 (Nm)	0.008	3 months	194.5 ± 72.2 (Nm)	0.125
6 months	220.7 ± 67.5 (Nm)	<0.001	6 months	165.9 ± 80.4 (Nm)	0.358
HHS			JHEQ		
Preoperative	25.2 ± 16.6		Preoperative	26.6 ± 11.3	
1 month	49.6 ± 25.3	<0.001	1 month	41.6 ± 17.5	<0.001
3 months	51.6 ± 25.6	<0.001	3 months	42.0 ± 20.0	<0.001
6 months	46.8 ± 27.2	<0.001	6 months	39.4 ± 19.7	<0.001
VAS					
Preoperative	75.5 ± 15.8				
1 month	41.2 ± 25.3	<0.001			
3 months	42.1 ± 24.7	<0.001			
6 months	46.5 ± 27.9	<0.001			

**Fig. 4:**
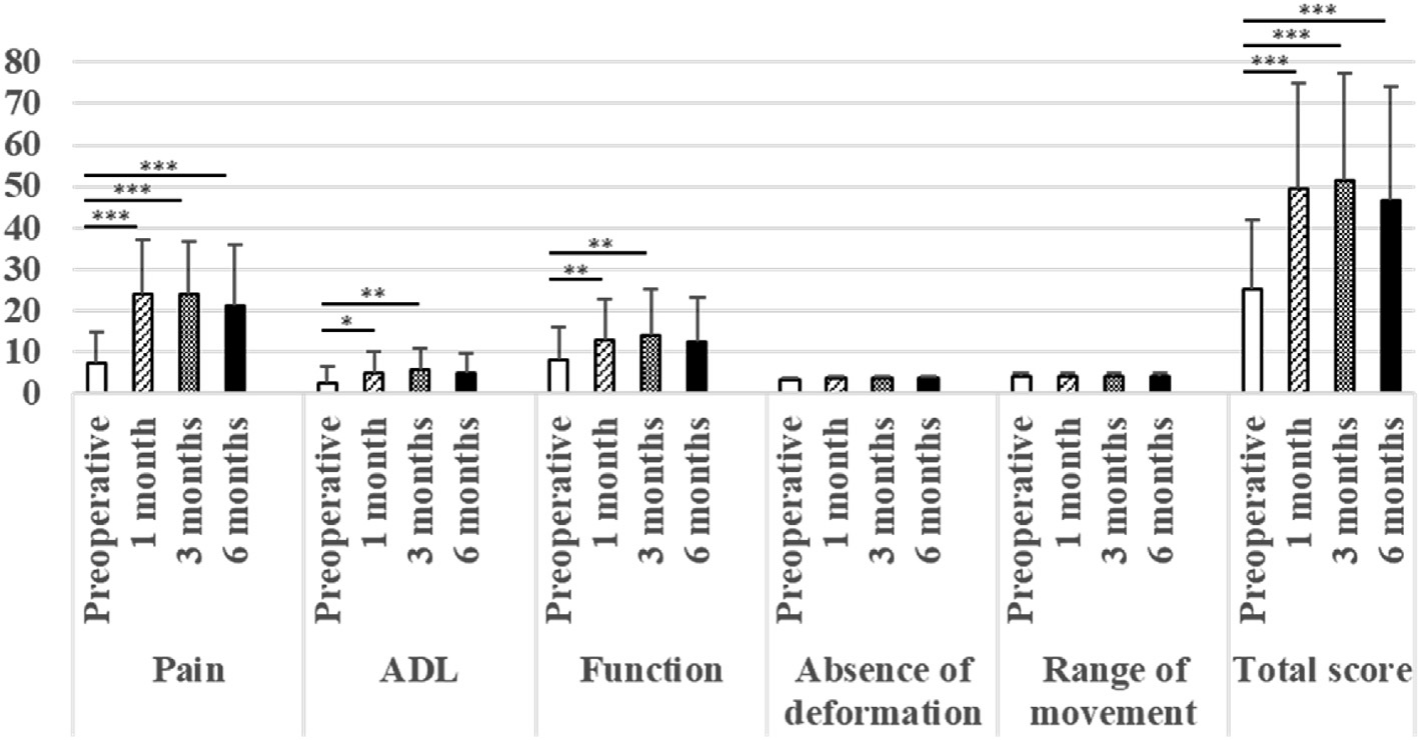
The Harris Hip score (HHS) and its subscale scores preoperatively and 1, 3, and 6 months postoperatively. The HHS ranges from 0 to 100. There are five subscales: pain (0–44 points); activities of daily living (ADL) (0–14 points); function (0–33 points); absence of deformity (0–4 points); and range of movement (0–5 points). ∗P<0.05. ∗∗P<0.01. ∗∗∗P<0.001.

**Fig. 5:**
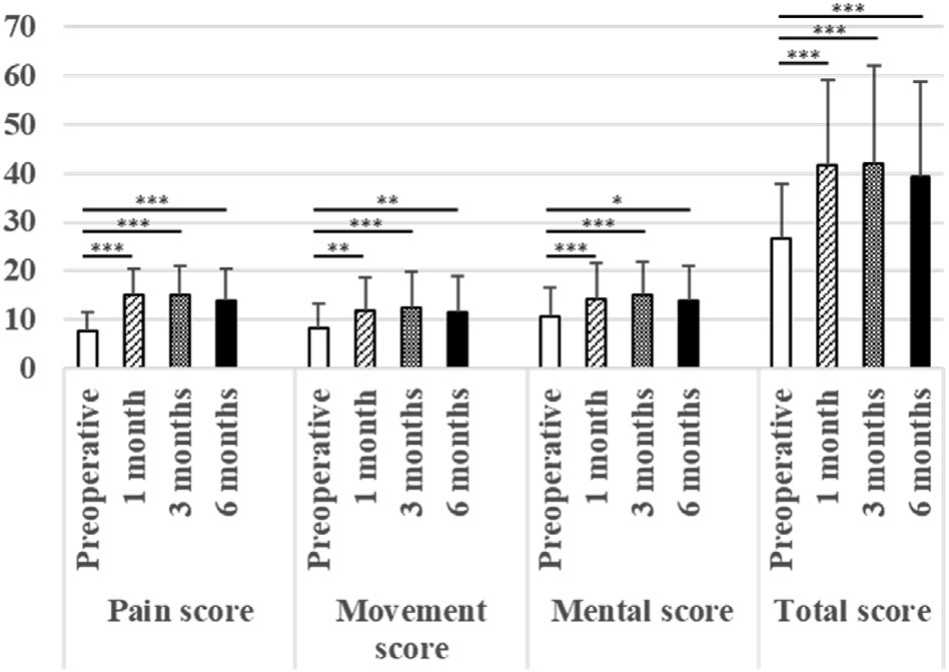
The Japanese Orthopaedic Association Hip Disease Evaluation Questionnaire (JHEQ) score and its subscale scores preoperatively and 1, 3, and 6 months postoperatively. The JHEQ score ranges from 0 to 84. There are three subscales: pain (0–28 points); movement (0–28 points); and mental health (0–28 points). ∗P<0.05. ∗∗P<0.01. ∗∗∗P<0.001.

The HHS, JHEQ, and VAS scores were significantly better at 1, 3, and 6 months postoperatively compared to those preoperatively (Table 2). The HHS subscales showed that the pain scores were significantly improved at 1, 3, and 6 months postoperatively compared to those preoperatively, and that the activities of daily living and functional scores were significantly higher at 1 and 3 months postoperatively compared to those preoperatively (Fig. 4). The pain, motor, and mental health JHEQ subscale scores were significantly increased at 1, 3, and 6 months postoperatively compared to those preoperatively (Fig. 5).

**Fig. 6:**
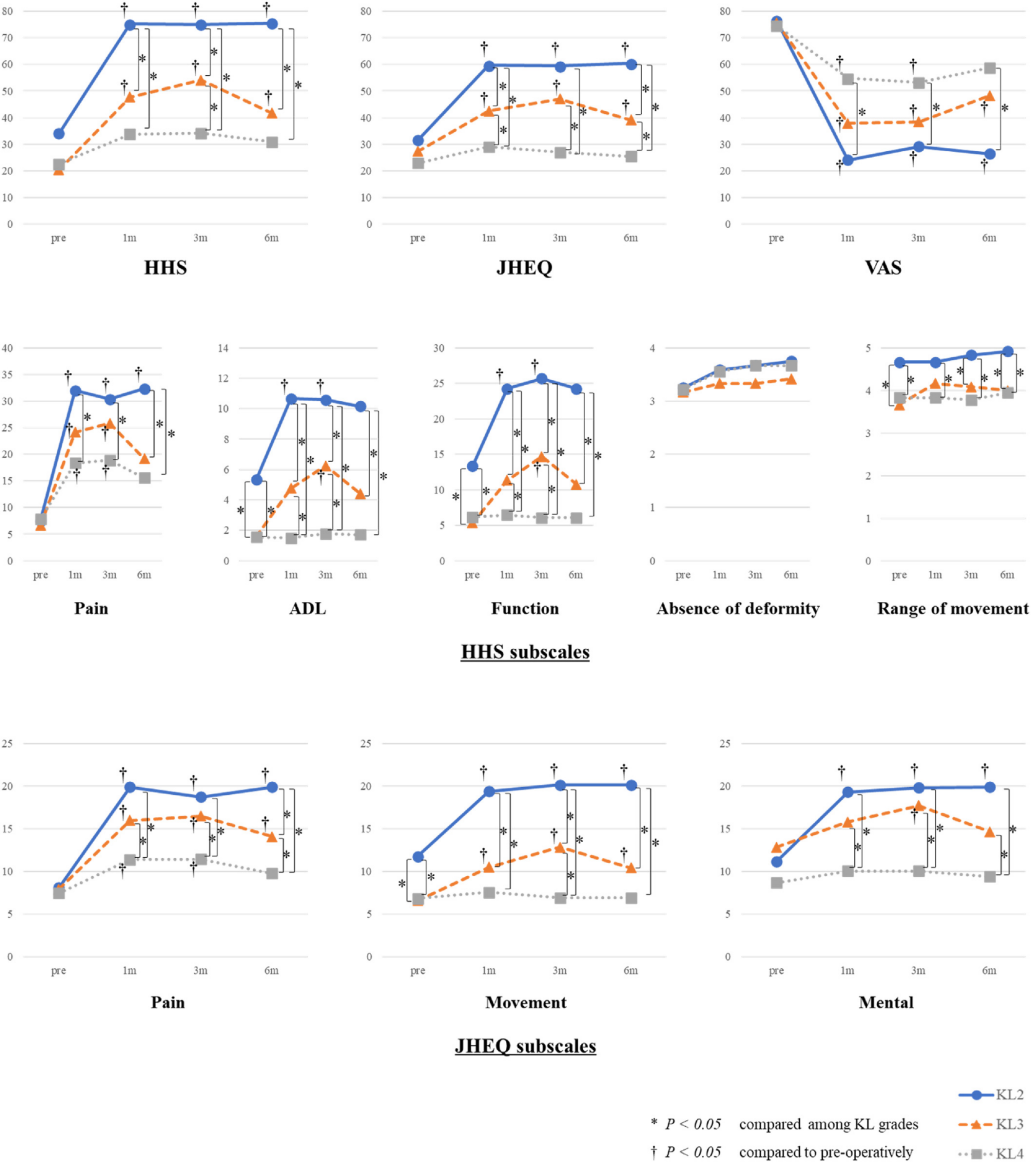
Harris Hip score (HHS), Japanese Orthopaedic Association Hip Disease Evaluation Questionnaire (JHEQ) score, visual analog scale (VAS) score, HHS subscale scores, and JHEQ subscale scores preoperatively and at 1, 3, and 6 months postoperatively for each KL grade. ∗P<0.05, compared among KL grades. †P<0.05, compared with preoperatively.

KL grades II and III had significantly better HHS, JHEQ, and VAS scores at 1, 3, and 6 months postoperatively than preoperatively; KL grade IV had significantly lower VAS scores at 1 and 3 months postoperatively than preoperatively. However, other scores were not significantly different between pre- and postoperatively. There were no significant differences in preoperative HHS, JHEQ score, and VAS score between KL grades, respectively. However, these scores at 1, 3, and 6 months postoperatively were better in the lower KL grades than in the higher grades. Similarly, for the HHS subscales of pain, ADL, and function, and the JHEQ subscales of pain, movement, and mental, KL II and III showed significant improvement in scores at almost all times postoperatively from preoperatively, while KL IV showed significant improvement only on the pain scale at 1 and 3 months postoperatively. When comparing KL grades, these scores were likewise better for the lower KL grades than for the higher KL grades (Fig. 6).

### 3.3 Imaging evaluation

The preoperative mean center edge angle and acetabular head index were not significantly different from those at 6 months postoperatively. In addition, the preoperative mean T2 mapping values of the femoral head and acetabulum were not significantly different from those at 6 months postoperatively at all sites (Table 3).

**Table 3:** Imaging evaluation results. CE angle; center edge angle, AHI; acetabular head index, Fa; femoral head part, Fc; femoral head center part, Fp; femoral head posterior part, Aa; acetabular anterior part, Ac; acetabular center part, Ap; acetabular posterior part.

CE angle	Mean ± S.D.	*P*	AHI	Mean ± S.D.	*P*
Preoperative	17.2 ± 12.8 (°)		Preoperative	69.0 ± 11.6	
6 months	16.9 ± 12.9 (°)	0.245	6 months	68.0 ± 12.1	0.175
T2 mapping value					
Fa			Aa		
Preoperative	58.5 ± 7.3		Preoperative	58.5 ± 9.5	
6 months	60.5 ± 8.3	0.181	6 months	58.9 ± 9.7	0.798
Fc			Ac		
Preoperative	57.0 ± 7.4		Preoperative	55.6 ± 7.9	
6 months	59.4 ± 9.3	0.157	6 months	58.9 ± 10.3	0.073
Fp			Ap		
Preoperative	57.1 ± 5.4		Preoperative	53.3 ± 6.2	
6 months	57.8 ± 9.0	0.785	6 months	55.5 ± 8.8	0.199

### 3.4 Safety evaluation

No deaths or severe life-threatening adverse events occurred during the 6-month follow-up period after the SVF cell injection. Five patients (11.9%) complained of mild hip pain, which lasted several days after surgery, but all resolved within a week. No local heat or infection of the hip occurred, and no patient required additional surgery, such as total hip arthroplasty, during the follow-up period.

## 4 Discussion

The most important finding of this study was that several clinical parameters, including the HHS, JHEQ score, and VAS score, improved significantly after the intra-articular injection of SVF cells in the hip joints of patients with OA. However, contrary to our hypothesis, SVF therapy did not improve the imaging results of the articular cartilage of the hip joint. To the best of our knowledge, this is the first prospective study to investigate the clinical and imaging results of such injections for hip OA treatment. These findings indicate that the intra-articular injection of SVF cells in the hip joint is a safe and favorable treatment that reduces hip OA symptoms.

SVF cells, in contrast to adipose tissue-derived MSCs and bone marrow-derived MSCs, are advantageous because they can be used rapidly for treatment without culturing. Notably, several studies have indicated the efficacy of SVF cell therapy for knee OA [27,28]. One study reported that intra-articular SVF cell injections for knee OA provided better pain relief and clinical outcomes than hyaluronic acid administration [16]. Another study showed that SVF was superior to platelet-rich plasma for reducing pain associated with knee OA from 1 month to 1 year postoperatively [29]. This may be because SVF cells promote inhibitory macrophages and T-regulatory cells, which have stronger pain-relieving effects because of reduced inflammatory markers [30,31]. Experiments involving animal models have confirmed the anti-inflammatory effects of SVF on knee OA [32,33]. SVF treatment of knee OA is being established as a safe and effective method; however, only a few studies showing the efficacy of SVF treatment for hip OA have been published. One previous study involved only six patients [34], and another was a large clinical trial that did not include a detailed clinical evaluation [35]. However, during this study, a prospective, varied, and detailed clinical evaluation was conducted using a sufficient sample size.

During this study, the HHS, JHEQ, and VAS scores at 1, 3, and 6 months postoperatively were significantly superior to those preoperatively. The HHS is a viable and credible tool for estimating outcomes after hip treatment, with clinically significant minimal differences of 4–10 points [36]. Furthermore, the HHS significantly improved from 25.2 preoperatively to 49.6, 51.6, and 46.8 at 1, 3, and 6 months postoperatively, respectively; therefore, its clinical effectiveness has been established. The pain subscale score significantly improved at 1, 3, and 6 months postoperatively, as did those of the activities of daily living subscale and functional subscale at 1 and 3 months postoperatively. In contrast, the absence of deformation and the ROM scores did not improve. One study that compared SVF cells, platelet-rich plasma, hyaluronic acid, and other treatments for knee OA showed that SVF cells resulted in the best improvement in pain scores postoperatively; however, the superiority of the Western Ontario McMaster Universities Arthritis Index, an optimal tool for clinically assessing the knee, was inconsistent among these treatments [29]. This indicates that SVF cells may be more beneficial for reducing pain than for improving joint function, and our results support this finding. All scores of the JHEQ pain, movement, and mental health subscales were significantly better at 1, 3, and 6 months postoperatively compared to those preoperatively. Because improvement in pain and joint function after therapy of the lower extremities is related to improvement in depression and anxiety symptoms [37], the pain relief provided by SVF cell treatment positively impacts mental health. Additionally, SVF cell therapy has been shown to improve the exercise function of Asian patients.

Regarding the KL classifications, the clinical scores after SVF cell therapy were generally significantly better for patients with lower grades than those with higher grades. We previously reported that SVF cell injections for knee OA result in superior clinical outcomes when the degeneration is less advanced [28]. Additionally, intra-articular injections of hyaluronic acid in the hip joint are more effective for lower KL grades [4]. Our results are consistent with those above, suggesting that SVF cell therapy for hip OA might have relatively better therapeutic effects on patients with less articular degeneration.

T2 mapping is an imaging technique that quantitatively assesses the degree of articular cartilage degeneration by detecting changes in the water and collagen content [23]. During the present study, no obvious improvement was observed in the T2 mapping values. During our previous study of SVF cell administration for knee OA, the T2 mapping values of the anterolateral tibia and anterolateral and posterolateral femurs were significantly improved at 6 and 12 months postoperatively compared to those preoperatively for patients with varus knee OA [28]. This indicated that articular cartilage degeneration improved in areas without mechanical stress. In contrast, the T2 mapping values did not improve significantly in other areas with high mechanical stress. Because most areas of the hip joint are weight-bearing zones, the T2 mapping values did not improve significantly during this study; this result was similar to that observed in the knee weight-bearing zones.

This study had several limitations. First, no control group was included. In the future, we plan to compare the intra-articular injection of SVF cells and that of other agents, such as hyaluronic acid and steroids. Second, clinical and imaging evaluations were performed for a short period (up to 6 months postoperatively); therefore, further investigations with longer follow-up periods are required. Third, the relationship between the number of intra-articular SVF cell injections and clinical outcomes was not evaluated. Fourth, the association between bone marrow lesions and bone cysts on MRI and the efficacy of SVF was not investigated. These bone lesions can be closely related to SVF outcomes; however, bone marrow lesions take approximately 9 months to resolve. Therefore, 6 months of postoperative observation is insufficient to determine the therapeutic effect of SVF on bone marrow lesions [38]. Finally, a larger number of patients is necessary to demonstrate the significance of SVF treatment based on the KL grade.

## 5 Conclusions

SVF cell therapy for hip OA was safely performed without complications, resulting in good short-term clinical outcomes. Therefore, we propose that intra-articular SVF cell injection in the hip joint is an innovative and effective treatment for patients with hip OA.

## Declarations of conflict interest

The authors declare that they have no conflict of interest.
